# Supplementary material for: Frequency-dependent decoupling of domain-wall motion and lattice strain in bismuth ferrite
Source: Nat Commun. 2018 Nov 22;9:4928. doi: 10.1038/s41467-018-07363-y (PMC6250669; doi:10.1038/s41467-018-07363-y)
Supplement: Supplementary file 1 — Supplementary Information [file 41467_2018_7363_MOESM1_ESM.pdf]

Supplementary information

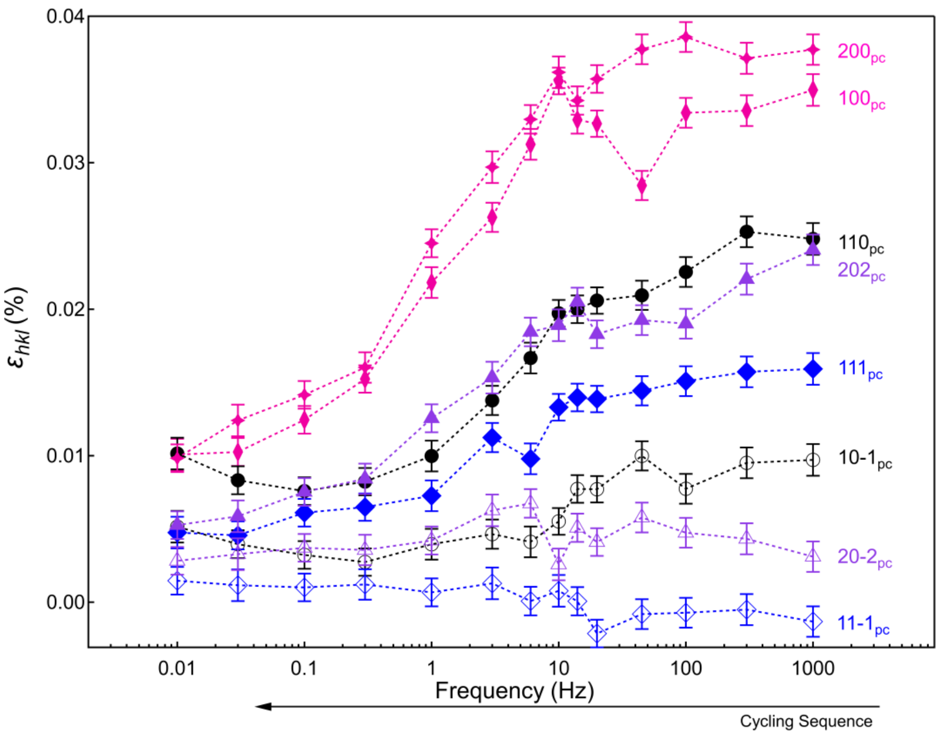

Supplementary Figure 1 Lattice strain calculated for individual reflections as a function of frequency. The lattice strain, ε_{hkl}, is calculated from diffraction peak shifts during unipolar field cycling with the scattering vector parallel to the electric field vector. The errors of the lattice strain arise from sinusoidal curve fitting of the lattice spacing responses during application of sinusoidal driving fields. Individual lattice strain is in general decreasing with decreasing frequency with a deviation at frequencies ~10 Hz. In $\{100\}_{pc}$ grains, this decrease can be understood due to the effective field decrease with decreasing frequency, while in $\{111\}_{pc}$ grains where the effective field increases with decreasing frequency, the strain of the grain is dominated by domain switching as presented in the main text. The reduction in 111_{pc} lattice strain shown in this figure is likely due to the increased domain switching response of these grains. At reducing frequency, the field in $\{111\}_{pc}$ grains increases and the domain switching fraction increases, thus 111_{pc} lattice plane experiences a compression stress due to volume constraint of such domains and 111_{pc} lattice strain reduces. Similar behaviour has been reported previously in a composition of (1-x)Ba($Zr_{0.2}Ti_{0.8}$)$_3$-x($Ba_{0.7}Ca_{0.3}$)TiO_3 [1]. The minor increase of $11\bar{1}_{pc}$ lattice strain with decreasing frequency is likely due to a convolution of the change in effective field strength, the compression stress in such domains and any grain-to-grain coupling from all other orientations.

Supplementary Table 1 Phase angle of lattice strain at different cycle numbers. During the measurements, data were collected over multiple cycles of the driving electric field. In order to evaluate stability of the sample's strain response at selected frequencies, the phase angle of 200_{pc} lattice strain, $\tan\delta_{200}$, was extracted for each cycle. The observed negative phase angle was found to be stable during the measurements.

Cycle number of the driving field	$\tan\delta_{200}$, 0.1 Hz driving field	$\tan\delta_{200}$, 0.3 Hz driving field	$\tan\delta_{200}$, 1 Hz driving field
cycle #1	-0.1212	-0.22108	-0.2573
cycle #2	-0.1215	-0.22116	-0.2573
cycle #3	-0.1215	-0.22119	-0.2573
cycle #4	-	-0.22116	-0.2570
cycle #5	-	-0.22114	-0.2570
cycle #6	-	-0.22116	-0.2570
cycle #7	-	-0.22116	-0.2570
cycle #8	-	-	-0.2570
cycle #9	-	-	-0.2570
cycle #10	-	-	-0.2570

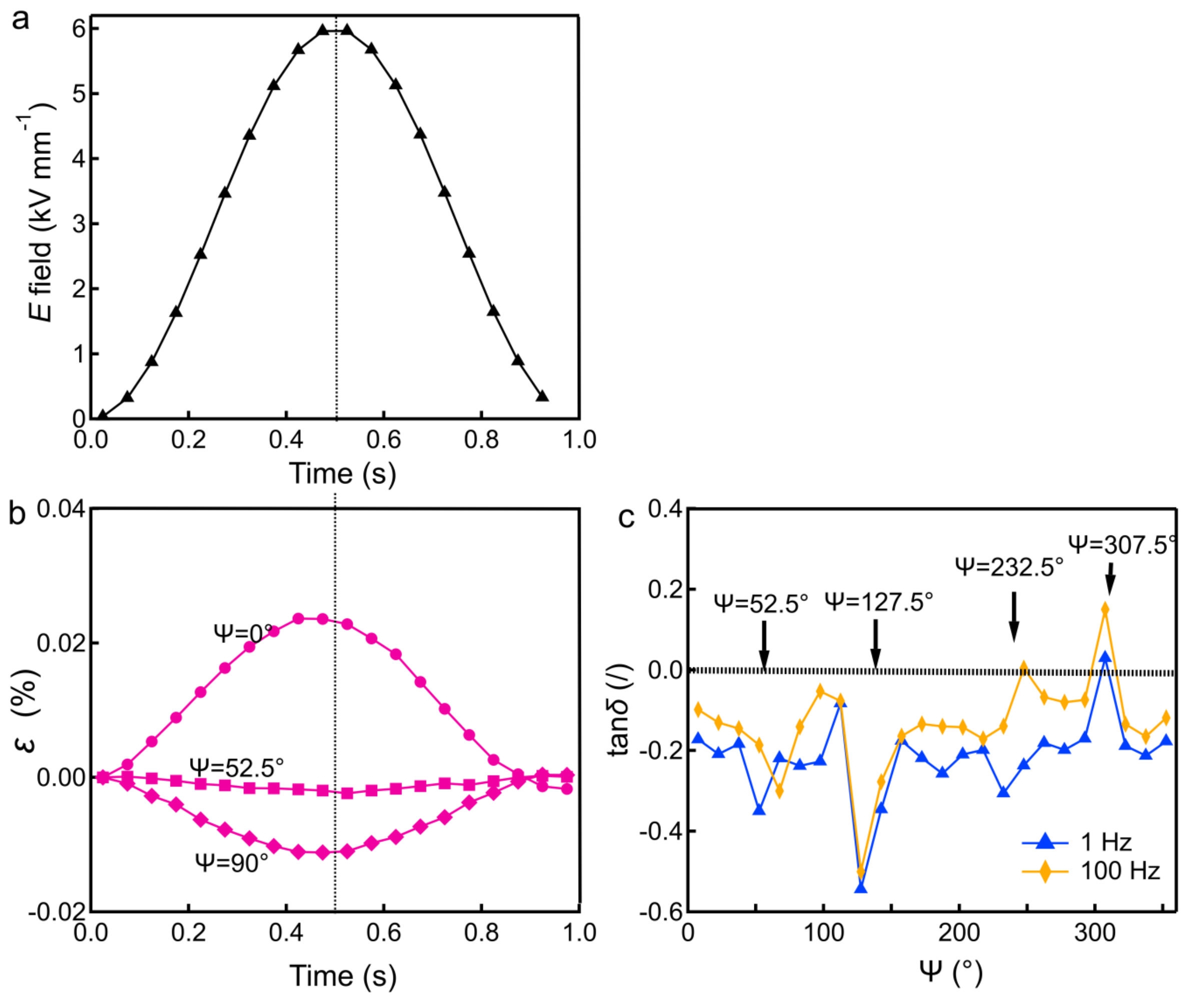

Supplementary Figure 2 Orientation-dependent phase angle of lattice strain. (a) Applied 6 kV mm^{-1} unipolar sinusoidal electric field, *E*, of 1 Hz, (b) resulting lattice strain response of 200_{pc} reflection, ε, at different angles to the applied electric field (0˚ means that the diffraction vector, **q**, of $\{200\}_{pc}$ grains is parallel to the field vector, **E**, while 90˚ means that is perpendicular to the electric field), and (c) tangent of the phase angle, tanδ, of the lattice response at different orientations with respect to the electric field vector, **E**, at 1 Hz and 100 Hz (note that the arrows indicate the sections where the fitting error is greater since the lattice strain is approximately zero as shown in panel b). It is obvious from panel (b) that at ~ 52.5˚ the lattice strain magnitude is approximately zero as has been described using a micromechanical model by Hall *et.al.* [2], while at lower **q**-**E** angles (e.g., 0°) the lattice strain is positive and at higher **q**-**E** angles (e.g., 90°) the lattice strain is negative. It is worth noting that at all orientations (disregard the sections with larger errors as indicated by black arrows in panel c), the peak values of lattice strain appear before the electric field peak (panel b, black dashed line), indicating phase leading of lattice strain at all orientations. This corresponds to negative strain-field phase angle as shown in panel (c) at both low and high frequencies (e.g., 1 Hz and 100 Hz), showing that the negative phase angle of lattice strain occurs in multiple grain orientations.

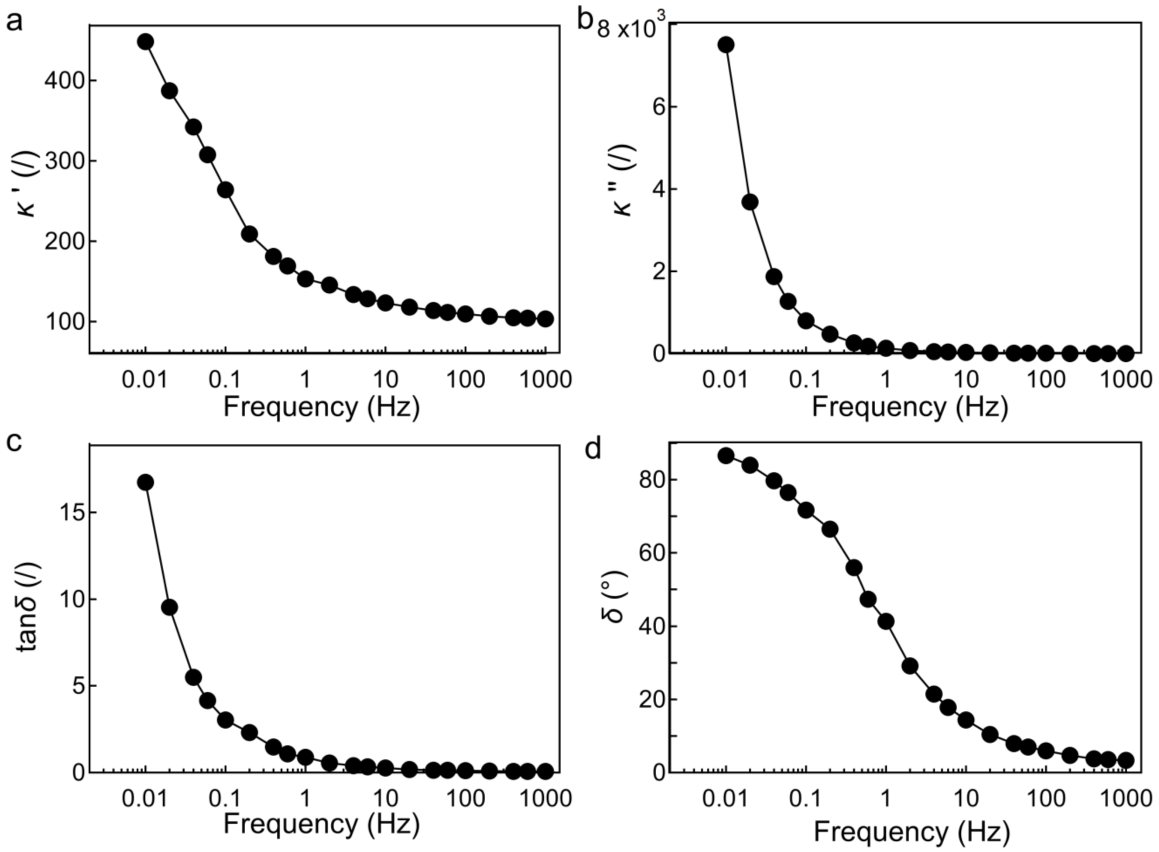

Supplementary Figure 3 Dielectric permittivity measured by unipolar 6 kV mm^{-1} field amplitude. This measurement is conducted at the same condition as the in situ X-ray diffraction (XRD) experiments on a sample from the same batch: (a) real part (κ'), (b) imaginary part (κ'') of the permittivity, (c) tangent of phase angle (tanδ; dielectric loss), and d) phase angle in degrees (δ) as a function of the driving frequency. The real (κ'), imaginary (κ'') part of the permittivity and dielectric loss (tanδ) (panel a-c) show a dispersive trend with decreasing driving frequency. Note that the change of κ'' with frequency (from 6 at 1000 Hz to 7500 at 0.01 Hz) is an order of magnitude larger than that of κ'(from 100 at 1000 Hz to 450 at 0.01Hz). The total κ'' can be represented by $\kappa''= \kappa_0 \cdot \kappa_d''(\omega) + \frac{\sigma_0}{\omega}$, where $\kappa_{0,}$ $\kappa_d''(\omega)$, σ_0 and ω are vacuum permittivity, frequency-dependent dielectric loss, specific bulk electrical conductivity and angular frequency, respectively [3]. Therefore, at low frequencies, κ'' is controlled by the bulk electrical conductivity of the sample ($\frac{\sigma_0}{\omega}$ term) as expected at such large driving fields (6 kV mm^{-1}). This is clearly seen by analysing the charge-voltage phase angle. The phase angle, δ, as a function of frequency (panel d) indeed shows an evolution from ~0° (capacitive response) at the high frequency end (1000 Hz) towards 90° (resistive/conductive response) at the low frequency end (0.01 Hz). Typical features of Maxwell-Wagner-like behaviour (step-like dispersion in $\kappa'$and local peaks in κ'' and tanδ) depend on measuring parameters and material properties and may not be visible under all conditions; in this case they are probably masked by the dominating contribution from the bulk electrical conductivity at 6 kV mm^{-1} field. Indeed, Maxwell-Wagner-like behavior clearly emerges in the dielectric response measured at weak fields (0.02 kV mm^{-1}), which is shown next in Supplementary Figure 4.

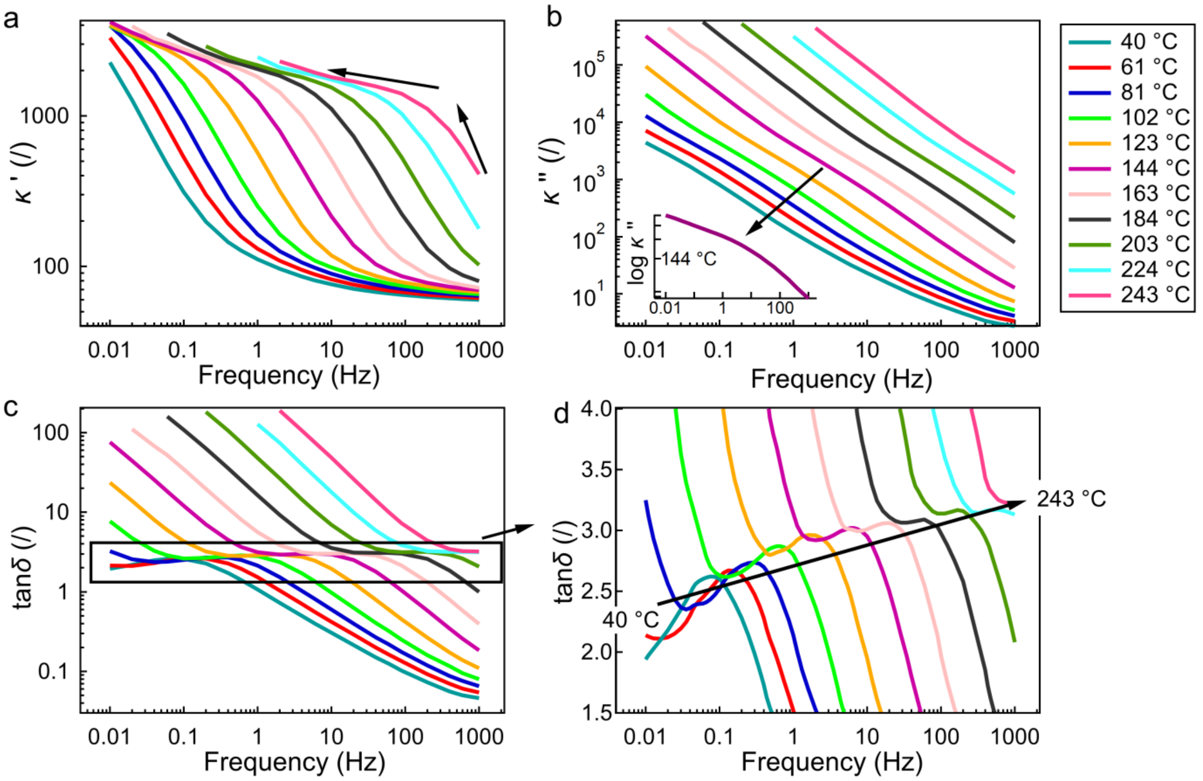

Supplementary Figure 4 Dielectric permittivity measured by bipolar 0.02 kV mm^{-1} field amplitude. This measurement is conducted with 0.02 kV mm^{-1} bipolar cyclic electric field on $BiFeO_3$ sample processed at the same condition as that used for in situ experiments [4]: (a) real part (κ'), (b) imaginary part (κ'') (Inset b: an enlarged view of the imaginary part at 144 ˚C on a logarithmic scale, indicating a weak and broad peak), and (c) tangent of phase angle as a function of frequency. Panel (d) shows an enlarged view of the tangent of phase angle to highlight the phase angle peaks at different temperatures as indicated by the black arrow. These data are consistent with behaviors expected for a Maxwell-Wagner-like dielectric response. A step-like increase with frequency in κ'(panel a, arrows) is obvious for all temperatures. This step increase is associated with a weak and broad peak in κ''(panel b), highlighted in the inset of panel b. The barely visible weak/broad peak in κ'' implies that this peak is probably buried into a high bulk conductivity background. More evident peaks are observed in the tangent of phase angle (tanδ) (panel c and d). The temperature dependence of all these features, i.e., κ'step-like behavior and $\kappa''$and tanδ peaks, showing a shift to higher frequencies with increasing temperature, confirms the thermally activated character of the Maxwell-Wagner mechanism, as would be expected since it is associated with conducting regions. Finally, after the step-like dispersion, it is noteworthy that κ' shows a further increase with lowering of the frequency, probably indicating Jonscher-type universal relaxation [5] due to hopping conductivity in the disorder system. The results are thus also consistent with hopping conductivity due to presence of electron holes as discussed previously for $BiFeO_3$ with p-type conductive character [6].

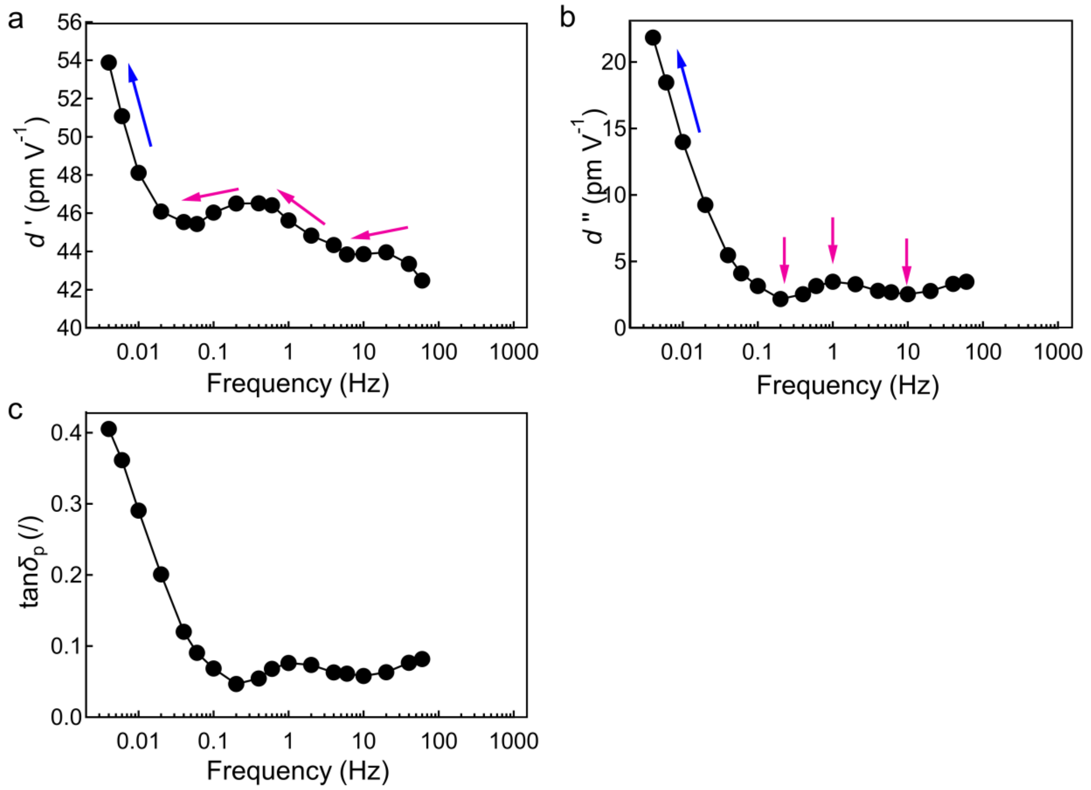

Supplementary Figure 5 Piezoelectric response measured by unipolar 6 kV mm^{-1} field amplitude. This measurement is conducted on a sample from the same batch at the same field as was used in in situ experiments: (a) real part (d'), (b) imaginary part (d''), and (c) phase angle ($\tan\delta_p$) of the piezoelectric response. In panel a and b, the magenta arrows indicate the decreasing and increasing of d' with decreasing frequency and the corresponding d'' peak with minima and maxima, while the blue arrows represent the strong dispersion in d' and d'' at the low frequency end, which is related to irreversible displacements of conducting domain walls [7]. The piezoelectric behaviour is consistent with the Maxwell-Wagner behaviour in permittivity as shown in Supplementary Figure 3. The peaks in the complex piezoelectric coefficients (d' and d'') and phase of the piezoelectric (strain-field) response ($\tan\delta_p$), observable in panel a-c, suggest Maxwell-Wagner-like behavior of the piezoelectric response [8]. In particular, the sequence of decreasing and increasing d' with decreasing frequency (panel a, magenta arrows), corresponding to the d'' peaks with minima and maxima (panel b, magenta arrows), suggests relaxation and retardation processes, respectively, consistent with the distinct characteristics of Maxwell-Wagner relaxation [8]. Note that relaxation and retardation processes were in the main manuscript for the first time directly identified in XRD analysis via the sign of the piezoelectric phase angle extracted for microscopic strains (negative – relaxation, positive – retardation; as explained in the manuscript). Finally, the strong dispersion in d' and d'' at the low frequency end (panel a and b, blue arrows) is related to irreversible displacements of conducting domain walls, in agreement with previous data on $BiFeO_3$ ceramics processed using different conditions with respect to the current sample [7]. Experimental data shown here are fully consistent quantitatively with the analytical model presented in the manuscript.

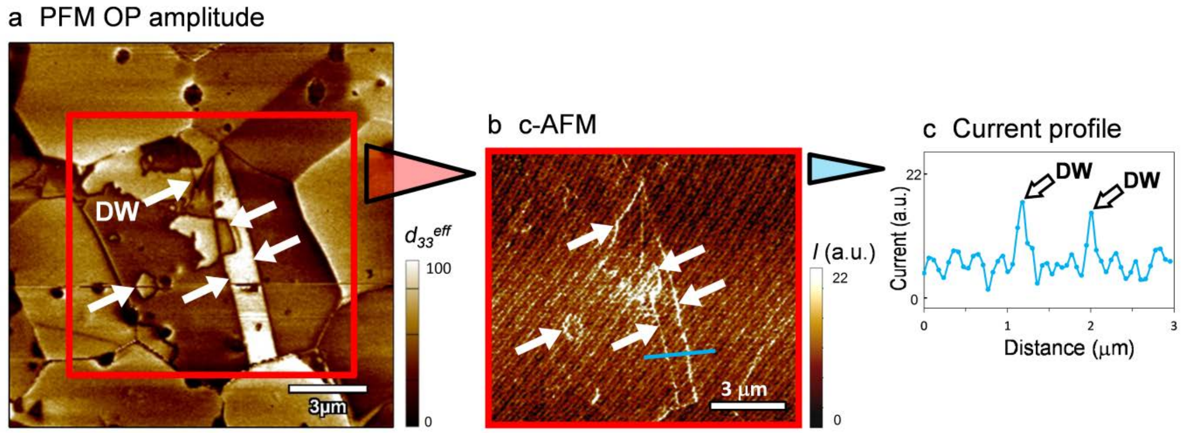

Supplementary Figure 6 Measurements of domain wall conductivity. (a) Out-of-plane (OP) piezoresponse force microscopy (PFM) amplitude image, (b) conductive atomic force microscopy (c-AFM) image of the area indicated with a box in panel (a), and (c) local electric-current profile across the blue line indicated in panel (b). The analysed sample is poled $BiFeO_3$ sintered at 780 °C (see methods in the main manuscript for details). The white arrows in respective images indicate domain wall positions. The regular, equally spaced parallel stripes in panel b are related to current signals representing artefacts which have no correlation with domain walls, grain boundaries (compare with PFM images) or any other topographical feature. PFM imaging was performed by applying to the tip 6 V of AC voltage, while c-AFM imaging was performed by biasing the tip with 22 V DC voltage. Additional details, including the measurement conditions and sample preparation can be found in [6,7]. The PFM image identifies different domain regions with domain walls separating them (arrows in panel a) inside grains. The c-AFM map shows that these domain walls are indeed associated with an enhanced electrical current (white lines in panel b marked with arrows, indicating increased current signal at domain walls). Additional evidence is provided by the electric-current profile crossing two domain walls (blue line in panel b) where two current peaks are clearly observed (panel c). These results confirm the higher electrical conductivity of domain walls in poled $BiFeO_3$ relative to that of the domains' interior (i.e., inner regions of the domain away from the domain walls). Since the orientation of these conductive domain walls is different in different grain families, it is reasonable to assume that the conductivity of individual grains in the direction of the external field axis will vary from grain to grain. This is the essential feature of our Maxwell-Wagner model.

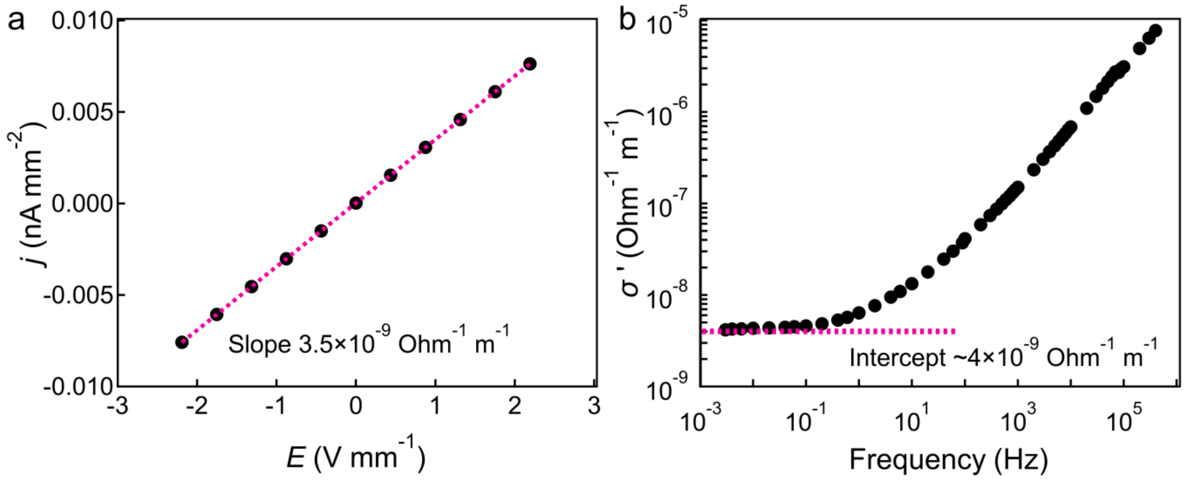

Supplementary Figure 7 Bulk electrical conductivity measurements. (a) Current-density–electric-field (j-E) curve and (b) real part of bulk electrical conductivity (σ') as a function of driving field frequency for $BiFeO_3$ ceramics (AC driving field 2.5 V mm^{-1}). To determine the specific bulk electrical conductivity of the $BiFeO_3$ sample, which were analysed by in situ XRD, DC (panel a) and AC (panel b) methods were used. The current-density–electric-field (j-E) curve in panel (a) shows a linear relationship, i.e., Ohm's law $j=\sigma_0 E$, where the slope represents the specific bulk electrical conductivity σ_0 of the sample. The specific bulk electrical conductivity using this classical j-E method is ~3.5×10^{-9} Ohm^{-1} m^{-1}. The real part of ac conductivity (σ') in panel (b) can be expressed as $\sigma' = \sigma_0 + \omega\kappa_0\kappa_d''(\omega)$, where σ_0, ω, κ_0 and $\kappa_d''(\omega)$ denote the specific bulk electrical conductivity, angular frequency, vacuum permittivity and frequency-dependent dielectric loss, respectively. It is seen in panel (b) that at low frequencies, σ' becomes frequency independent, levelling off at values corresponding to σ_0, i.e., $\sigma' \sim \sigma_0$ (intercept in panel b). The bulk electrical conductivity using this AC impedance method was thus determined as ~4×10^{-9} Ohm^{-1} m^{-1}. Within the limits of experimental methods used, the specific bulk electrical conductivity of $BiFeO_3$ determined with the two methods is consistent, showing values on the order of 10^{-9} Ohm^{-1} m^{-1}. These values were used as a reference in the analytical modelling presented in the main manuscript.

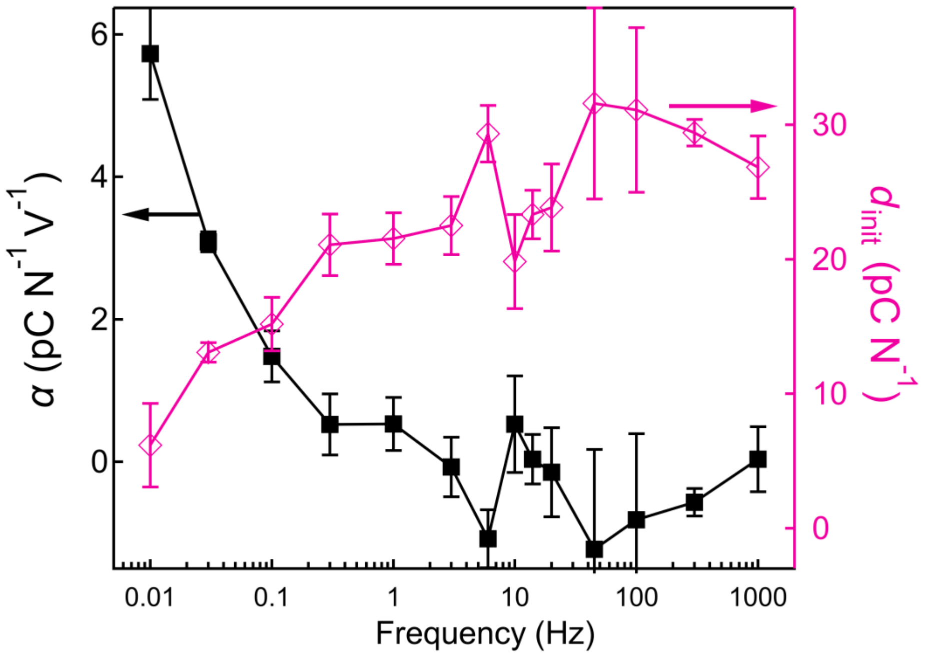

Supplementary Figure 8 Non-linearity in piezoelectric response of $BiFeO_3$. It is represented by Rayleigh reversible d_{init} and irreversible α parameter. In terms of the Rayleigh law describing piezoelectric response of ferroelectrics [9], the field-dependent, total piezoelectric d_{33} coefficient can be described by a linear term d_{init}, due to reversible displacements of the domain walls and intrinsic lattice piezoelectric response, and a field-dependent term αE_{max}, due to irreversible displacements. The total d_{33} can thus be expressed by $d_{33} = d_{\mathrm{init}} + \alpha E_{\mathrm{max}}$. The field-dependent piezoelectric coefficients of $BiFeO_3$, represented in the plot as d_{init} and α, were obtained experimentally with in situ XRD at different frequencies and field amplitudes. The errors arise from linear curve fitting on piezoelectric responses in the driving field range from 3 kV mm^{-1} to 7 kV mm^{-1}. The large non-linearity below ~ 0.3 Hz is shown by the significant increase from 0.5 to ~6 of the Rayleigh irreversible parameter α [9]. At low frequencies there is more irreversible domain wall motion, i.e., α increases at low frequencies. In contrast, d_{init}, which represents the reversible domain wall motion component and lattice strain, decreases.

Supplementary Table 2 Reproducibility of strain response during cycling. Lattice strain extracted from diffraction patterns and macroscopic strain values at the same frequency measured before and after the full frequency cycling for both unipolar and bipolar electric field. The errors of lattice strain and the macroscopic strain arise from sinusoidal curving fitting on these responses during application of sinusoidal driving field. The strain magnitude is generally the same within errors. These experiments ruled out crack formation and radiation damage under long-term exposure to the beam as dominant origins of the microscopic strains decoupling.

Driving electric field	100_{pc} lattice strain	200_{pc} lattice strain	Total lattice strain	Macroscopic Strain
Unipolar 20 Hz	0.033±0.003%	0.036±0.003%	0.018±0.002%	0.0169±0.0006%
Unipolar 20 Hz after 40 min cycling interval	0.028±0.003%	0.034±0.004%	0.016±0.002%	0.0171±0.0006%
Bipolar 1000 Hz	0.066±0.003%	0.067±0.003%	0.018±0.002%	0.0198±0.0009%
Bipolar 1000 Hz after 12 min cycling interval	0.064±0.003%	0.063±0.004%	0.018±0.002%	0.0182±0.0008%
Bipolar 20 Hz	0.062±0.003%	0.056±0.004%	0.016±0.002%	0.017±0.004%
Bipolar 20 Hz after 135 min cycling interval	0.070±0.003%	0.063±0.004%	0.017±0.002%	0.013±0.005%

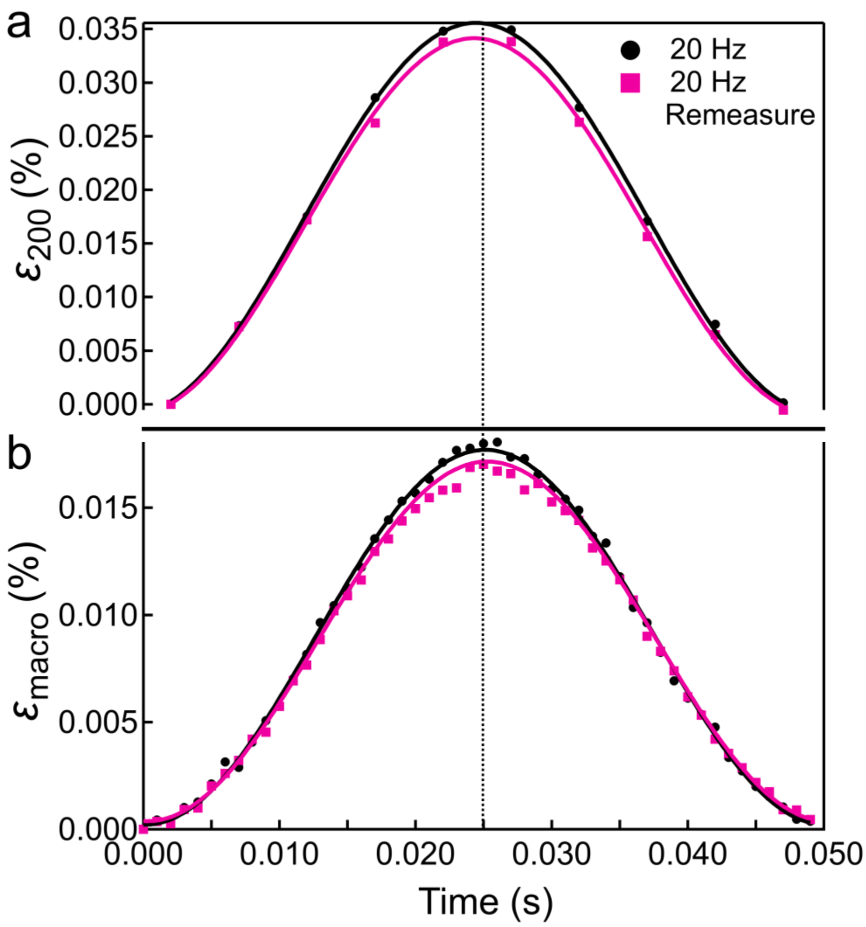

Supplementary Figure 9 Reproducibility of strain response. (a) 200_{pc} lattice strain and (b) macroscopic strain of the initial measurement at 20 Hz and repeated measurement after the full cycling sequence (from 1000 Hz to 0.01 Hz) at 20 Hz. As shown in (a), the initial measured amplitude of 200_{pc} lattice strain at 20 Hz (black circles) is 0.036±0.003%. This strain amplitude at 20 Hz during the repeated measurement (magenta squares) after the full cycling sequence (from 1000 Hz to 0.01 Hz) is 0.034 ± 0.004%, which is within the experimental error the same as that before the varying frequency measurements. The responses and errors are obtained from the shown sinusoidal curve fitting (black and magenta curves) on 200_{pc} lattice strain (black circles and magenta squares). Sinusoidal curve fitting is also employed on the macroscopic data to extract the macroscopic response and error as shown in panel b. In panel (b), the macroscopic strain of the initial measurement and repeated measurement for 20 Hz is also equivalent in magnitude, showing 0.0171%±0.0006% and 0.0177%±0.0006%, respectively. The result confirms that the strain magnitude at 20 Hz after the full frequency cycling sequence is not significantly changed on repeated measurements, suggesting that grain interactions in the $BiFeO_3$ ceramic are stable during sub-coercive field application. Interruption of grain-scale mechanical coupling, for example by sample cracking, is thus excluded as the main reason for decoupling of microscopic strain mechanisms.

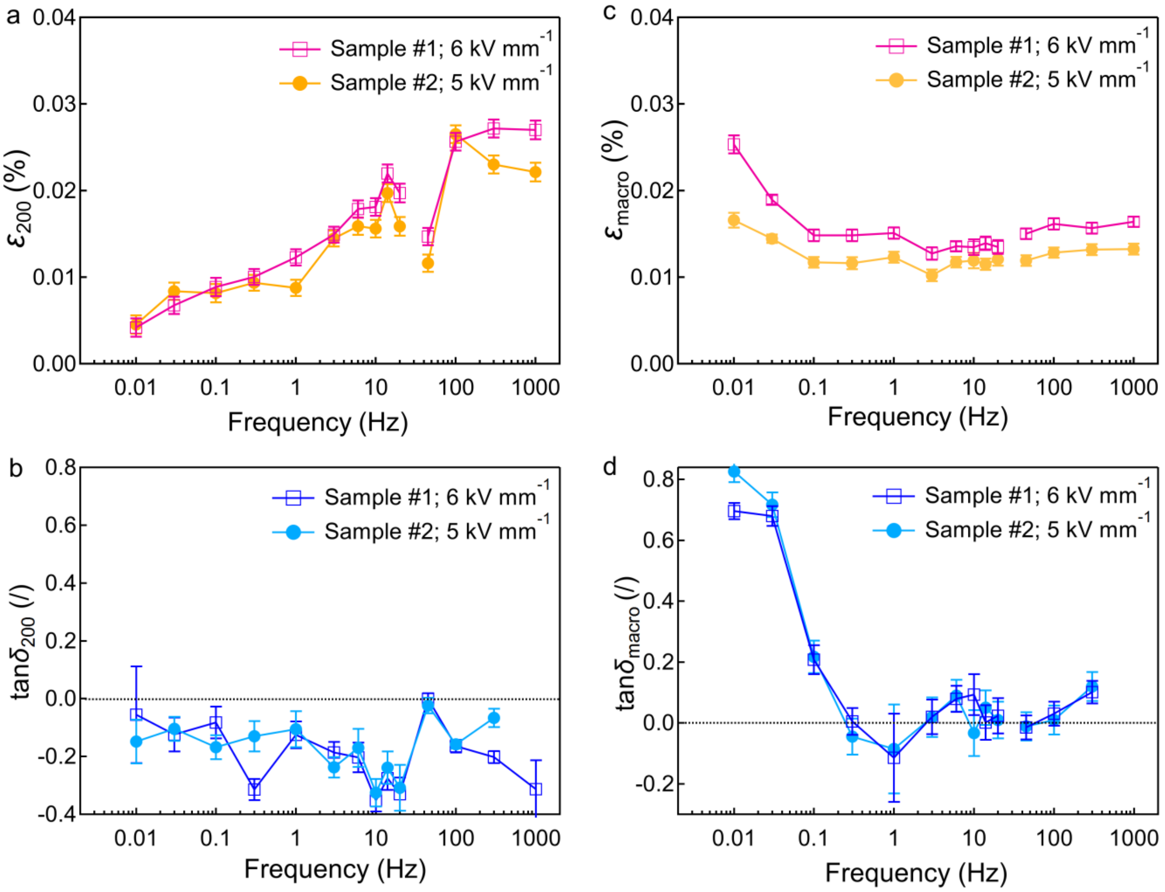

Supplementary Figure 10 Reproducibility of strain response. Two additional samples measured under electric field amplitudes of 5 kV mm^{-1} and 6 kV mm^{-1}: (a) lattice strain of 200_{pc} peak, ε_{200}; (b) phase angle of 200_{pc} lattice strain, $\tan\delta_{200}$; (c) macroscopic strain, $\varepsilon_{\mathrm{macro}}$ and (d) phase angle of macroscopic strain, $\tan\delta_{\mathrm{macro}}$. The errors arise from sinusoidal curve fitting on the lattice strain and macroscopic strains during application of sinusoidal cyclic field. The frequency dispersion of lattice strain, tangent of the phase angle of lattice strain, macroscopic strain and tangent of the piezoelectric phase angle of macroscopic strain between different samples are consistent. This confirms that the data obtained during full frequency cycling is reliable within experimental errors.

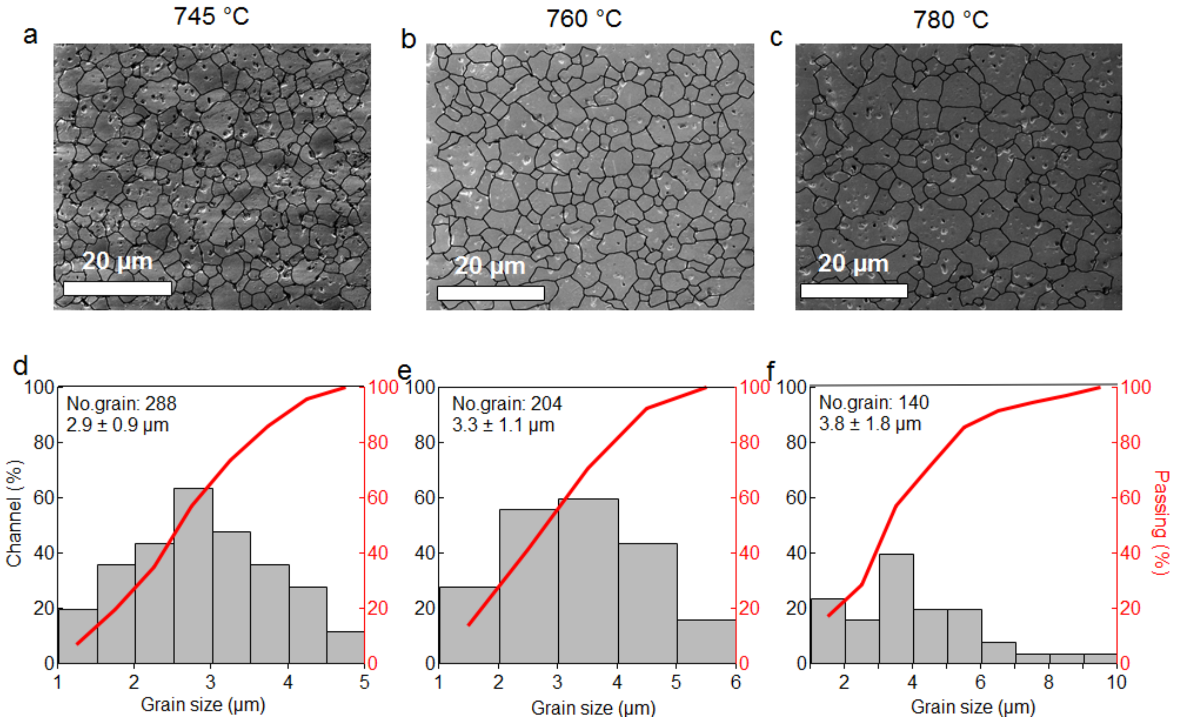

Supplementary Figure 11 Microstructural analysis. These samples are sintered at (a) 745 ˚C, (b) 760 ˚C, (c) 780 ˚C and d-f show their grain size distribution analysed from the SEM images, respectively. These samples are thermally-etched before taking SEM images. The grain boundaries were drawn on the images for analysis of the grain size. The grain size, expressed as the Feret's diameter mean values [10], analysed for these images (using the UTHSCSA ImageTool Software) are 2.9 ± 0.9 µm (panel d), 3.3 ± 1.1 µm (panel e) and 3.8 ± 1.8 µm (panel f) for the three samples, respectively. However, it is worth noting that there are occasional large grains (i.e., grains with diameter greater than ~ 7 µm; see histogram in panel f) in the sample sintered at 780 ˚C due to excessive grain growth at this highest sintering temperature. Small variations in the average grain size in our samples (between 2.9 and 3.8 µm) is expected considering the small maximum difference in the sintering temperature (35 °C). We note that the samples were sintered at such temperatures for the purpose of varying the grain size only slightly and thus verify reproducibility of XRD measurements for different processing conditions (i.e., sintering temperature). In particular, we were looking at the reproducibility of the unusual increase of lattice strain with increasing frequency in samples sintered at different temperatures but with comparable microstructure (we show subsequently in this SI material that this is confirmed in all three samples). Note that in contrast to other ferroelectrics such as PZT or $BaTiO_3$, the processing of $BiFeO_3$ ceramics is limited to a narrow temperature range (between ~750 and ~790°C) due to its thermodynamic instability, peritectic decomposition of Bi-rich phases and Bi_2O_3 sublimation [11].

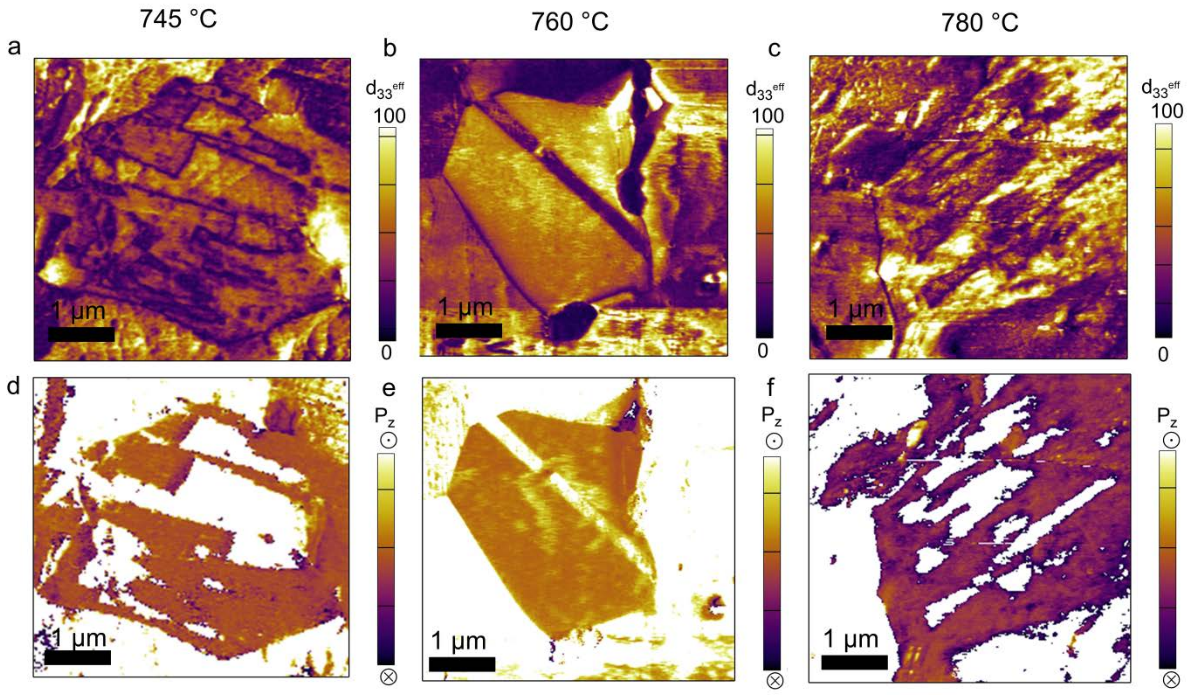

Supplementary Figure 12 Domain structures resulting from different sintering temperatures. (a-c) In-plane (IP) PFM amplitude and (d-f) phase images of $BiFeO_3$ samples sintered at 745 ˚C, 760 ˚C and 780 ˚C, respectively. P_z in panel d-f represents the direction of the polarization, i.e., dot in circle indicates the polarization point out of the paper plane, while cross in circle has a 180º phase difference and points into the paper plane. For PFM analysis, the samples were ground with SiC paper and polished with a diamond paste for ~2 h. Sample thickness ranged between 0.2 and 0.5 mm. PFM was performed on an area of 5 x 5 μm to inspect the domain structure. We note that the domain structure analysis was performed on poled samples (as such samples were indeed measured by in situ XRD) and were analysed by in-plane PFM imaging, rather than out-of-plane. The reason is that in poled samples the spontaneous polarization of the majority of domains is oriented out-of-plane, resulting in small out-of-plane PFM phase contrast (not shown here), making the analysis of domains difficult. The domains shown in this figure show regular, lamellar-like morphology, typically observed in $BiFeO_3$ ceramics [7]. No obvious qualitative difference is observed in the domain structure of the three samples sintered at different temperatures, consistent with their similar grain sizes (Supplementary Figure 11).

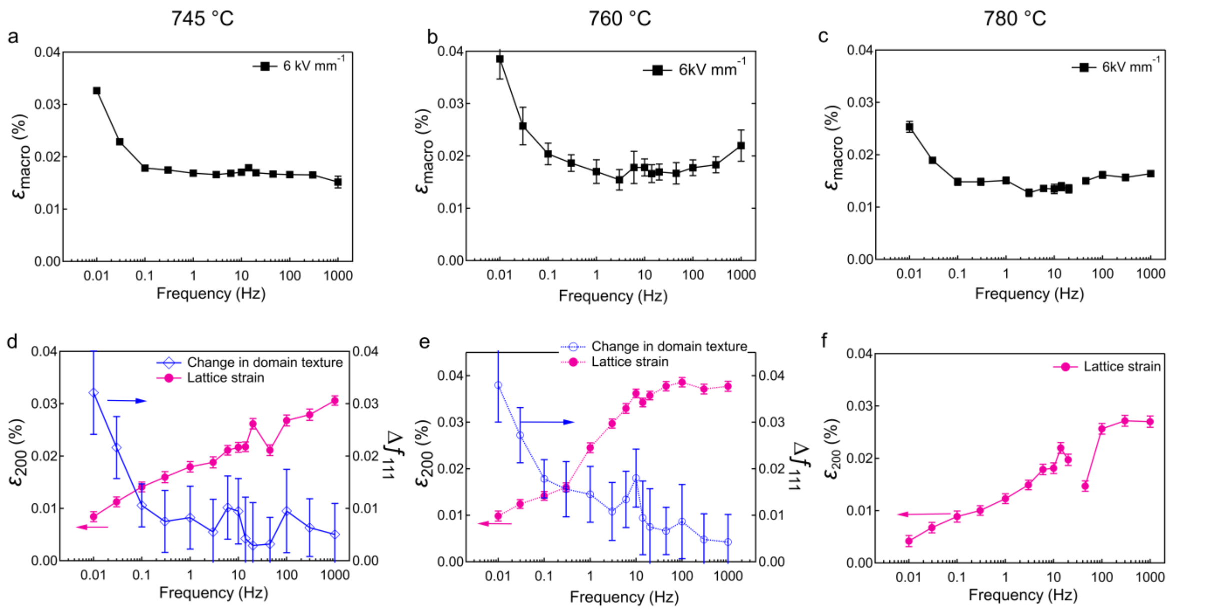

Supplementary Figure 13 Reproducibility of results for samples sintered at different temperatures. (a-c) Macroscopic strain, $\varepsilon_{\mathrm{macro}}$, and (d-f) microscopic domain texture, Δf_{111} (blue) and 200_{pc} lattice strain, ε_{200} (magenta) obtained from in situ XRD data of the $BiFeO_3$ samples sintered at 745 ˚C, 760 ˚C and 780 ˚C, respectively. The error bars of lattice strain, change in non-180 domain texture and macroscopic strain arise from sinusoidal curve fitting on these responses during application of sinusoidal cyclic electric field. The domain texture values had larger errors for the sample sintered at 780 ˚C due to the presence of bigger grains that impact the quality of the diffraction images, thus values are not shown for this sample. Slightly lower temperatures (745 ˚C and 760 ˚C) were chosen to reduce the grain size and thus improve sampling statistics of the XRD measurements. These specific temperatures were chosen to test the reproducibility of XRD measurements in samples with slightly different grain size. As shown in this figure, the frequency-dependent behaviour of samples sintered at 745 ˚C, 760 ˚C and 780 ˚C is qualitatively similar, showing a good reproducibility of the in situ XRD experiments. All the data confirm the increasing trend of lattice strain with increasing frequency.

Supplementary References

1 Ehmke, M. C. *et al.* Resolving structural contributions to the electric-field-induced strain in lead-free (1-x)$Ba(Zr_{0.2}Ti_{0.8})O_3$ - x$(Ba_{0.7}Ca_{0.3})TiO_3$ piezoceramics. *Acta Materialia* **66**, 340-348 (2014).

2 Hall, D. A., Steuwer, A., Cherdhirunkorn, B., Mori, T. & Withers, P. J. Analysis of elastic strain and crystallographic texture in poled rhombohedral PZT ceramics. *Acta Mater.* **54** 3075–3083 (2006).

3 Jonscher, A. K. Dielectric relaxation in solids. *Chelsea Dielectrics Press* (1983).

4 Rojac, T. *et al.* Piezoelectric response of $BiFeO_3$ ceramics at elevated temperatures. *Appl. Phys. Lett.* **109**, 042904 (2016).

5 Lunkenheimer, P., Fichtl, R., Ebbinghaus, S. G. & Loidl, A. Nonintrinsic origin of the colossal dielectric constants in $CaCu_3Ti_4O_{12}$. *Phys. Rev. B* **70**, 172102 (2004).

6 Rojac, T. *et al.* Domain-wall conduction in ferroelectric $BiFeO_3$ controlled by accumulation of charged defects. *Nature Mater.* **16**, 322-327 (2017).

7 Rojac, T., Ursic, H., Bencan, A., Malic, B. & Damjanovic, D. Mobile Domain Walls as a Bridge between Nanoscale Conductivity and Macroscopic Electromechanical Response. *Adv. Funct. Mater.* **25**, 2099-2108 (2015).

8 Damjanovic, D., Maeder, M. D., Martin, P. D., Voisard, C. & Setter, N. Maxwell–Wagner piezoelectric relaxation in ferroelectric heterostructures. *J. Appl. Phys.* **90**, 5708-5712 (2001).

9 Damjanovic, D. Stress and frequency dependence of the direct piezoelectric effect in ferroelectric ceramics. *J. Appl. Phys.* **82**, 1788-1797 (1997).

10 Walton, W. H. Feret‘s Statistical Diameter as a Measure of Particle Size. *Nature* **162**, 329–330 (1948).

11 Rojac, T. *et al.* $BiFeO_3$ Ceramics: Processing, Electrical, and Electromechanical Properties. *J. Am. Ceram. Soc.* **97**, 1993-2011 (2014).
